# Very Low Rates of Spontaneous Gene Deletions and Gene Duplications in *Dictyostelium discoideum*

**DOI:** 10.1007/s00239-022-10081-1

**Published:** 2022-12-09

**Authors:** Shelbi E. Gill, Frédéric J. J. Chain

**Affiliations:** Department of Biology, University of Massachusetts Lowell, Lowell, MA 01854-2874 USA

**Keywords:** *Dictyostelium discoideum*, Mutation, Mutation accumulation experiment, Mutation rate, GC content, Copy number variation

## Abstract

The study of spontaneous mutation rates has revealed a wide range of heritable point mutation rates across species, but there are comparatively few estimates for large-scale deletion and duplication rates. The handful of studies that have directly calculated spontaneous rates of deletion and duplication using mutation accumulation lines have estimated that genes are duplicated and deleted at orders of magnitude greater rates than the spontaneous point mutation rate*.* In our study, we tested whether spontaneous gene deletion and gene duplication rates are also high in *Dictyostelium discoideum*, a eukaryote with among the lowest point mutation rates (2.5 × 10^–11^ per site per generation) and an AT-rich genome (GC content of 22%)*.* We calculated mutation rates of gene deletions and duplications using whole-genome sequencing data originating from a mutation accumulation experiment and determined the association between the copy number mutations and GC content. Overall, we estimated an average of 3.93 × 10^–8^ gene deletions and 1.18 × 10^–8^ gene duplications per gene per generation. While orders of magnitude greater than their point mutation rate, these rates are much lower compared to gene deletion and duplication rates estimated from mutation accumulation lines in other organisms (that are on the order of ~ 10^–6^ per gene/generation). The deletions and duplications were enriched in regions that were AT-rich even compared to the genomic background, in contrast to our expectations if low GC content was contributing to low mutation rates. The low deletion and duplication mutation rates in *D. discoideum* compared to other eukaryotes mirror their low point mutation rates, supporting previous work suggesting that this organism has high replication fidelity and effective molecular machinery to avoid the accumulation of mutations in their genome.

**Supplementary Information:**

The online version contains supplementary material available at 10.1007/s00239-022-10081-1.

## Background

Mutations induce genetic variation that underlies the evolution of all organisms. The mutational rate and fitness effects of spontaneous mutations are therefore important to understand evolution and heritable diseases (Katju and Bergthorsson 2019). However, it is challenging to determine how often mutations spontaneously arise because mutations with deleterious fitness effects are expected to be purged from the genome within several generations by natural selection (Hall et al. 2013). As a result of this, the effect of selection must be minimized for an accurate estimate of the spontaneous mutation rate over many generations. One of the most powerful ways to directly assess mutation rates is via a mutation accumulation (MA) experiment in conjunction with whole-genome sequencing. MA experiments maintain multiple lineages at small population size and undergo repeated bottlenecks for multiple generations to accumulate spontaneous mutations under low levels of natural selection (Halligan and Keightley 2009). Whole-genome sequencing of MA lines can be performed to determine mutations that have accumulated during the experiment, which are then used to estimate the genome-wide rate and range of spontaneous mutations (Kondrashov and Kondrashov 2010).

**Fig. 1:**
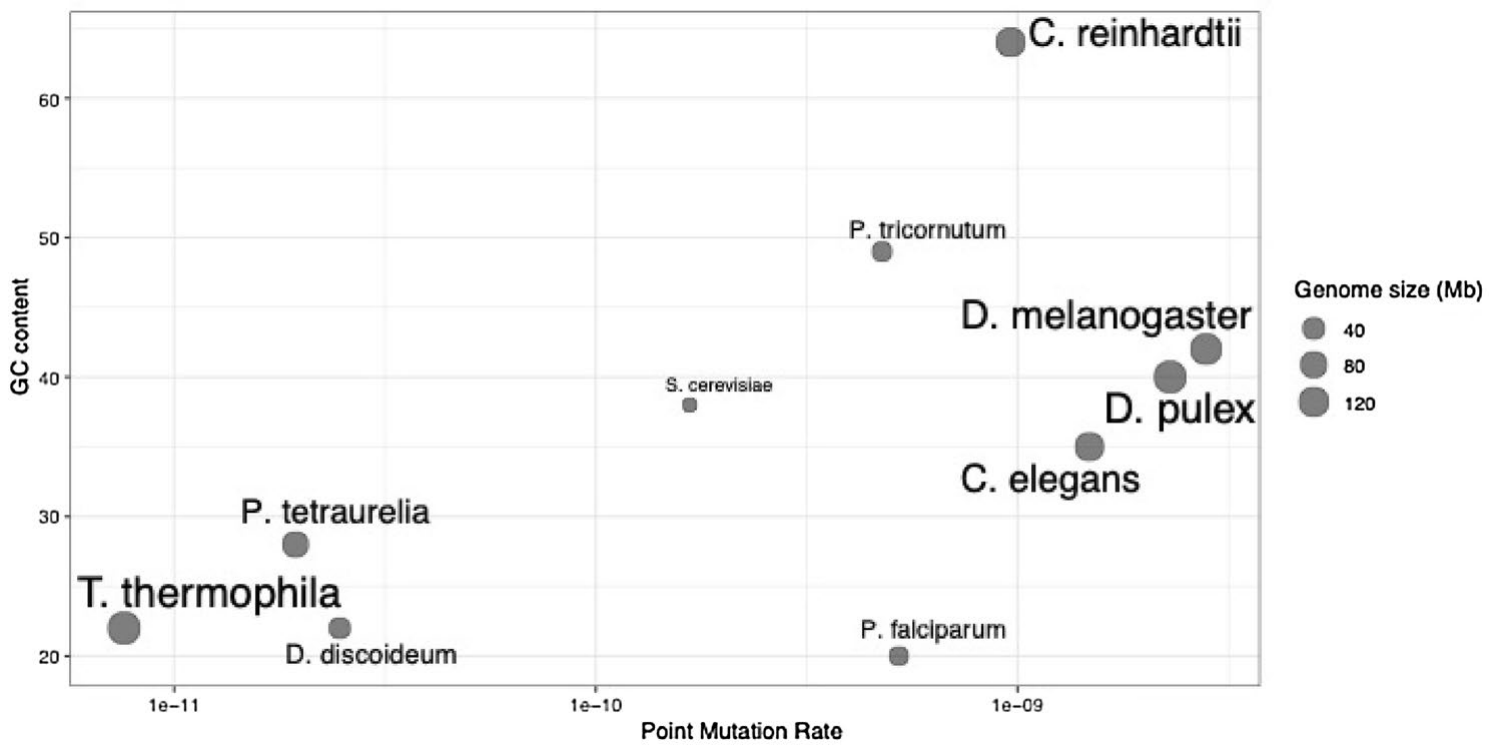
The relationship among genome-wide GC content, point mutation rate, and genome size. Select point mutation rates from mutation accumulation experiments are plotted along the x-axis (log_10_) with the corresponding genome GC content (y-axis) and genome size (proportional to bubble and text size)

Amongst eukaryotes, the single nucleotide mutation rates calculated from MA experiments range between ~ 8 × 10^–12^ to ~ 3 × 10^–9^ per site per generation. At the higher end of these point mutation rates are *Drosophila melanogaster* at 2.8 × 10^–9^ (Keightley et al. 2014), *Daphnia pulex* at 2.3 × 10^–9^ (Flynn et al. 2017), and *Caenorhabditis elegans* at 2.7 × 10^–9^ (Denver et al. 2009), as measured per nucleotide site per generation. In the middle of this range lies *Chlamydomonas reinhardtii* reported at 9.63 × 10^–10^ (Ness et al. 2012), *Phaeodactylum tricornutum* at 4.77 × 10^–10^ (Krasovec et al. 2019), *Plasmodium falciparum* at between 2.10—5.23 × 10^–10^ (Bopp et al. 2013; Hamilton et al. 2017; McDew-White et al. 2019), and *Saccharomyces cerevisiae* at 1.67 × 10^–10^ (Zhu et al. 2014). At the lower end of this range are *Tetrahymena thermophila* at 7.61 × 10^–12^ (Long et al. 2016), *Paramecium tetraurelia* at 1.94 × 10^–11^ (Sung et al., 2012), and *Dictyostelium discoideum* at 2.47 × 10^–11^ (Kucukyildirim et al. 2020). The variation in mutation accumulation in genomes is associated with a combination of factors that vary across taxa. Mutation rates and spectra are largely driven by the combined effects of DNA damage and repair (Volkova et al. 2020), which can differ by organism based on properties such as genome size and effective population size that influence the efficiency of selection on maintaining high DNA replication and repair fidelity and minimizing deleterious mutations (Lynch 2011, 2016; Kucukyildirim et al. 2020). In addition, mutation occurrence within a genome is affected by various genomic features such as GC content and CpG sites (Ananda et al. 2011; Hodgkinson and Eyre-Walker 2011; Schaibley et al. 2013). For example, CpG dinucleotide sites are highly mutable (Baer et al. 2007; Mugal and Ellegren 2011; Aggarwala and Voight 2016), partly attributed to cytosine methylation that promotes spontaneous deamination, substituting cytosines for thymines. However, the hypermutability of CpG sites has been observed even in the absence of cytosine methylation, suggesting alternative mechanisms driving high mutation rates in GC-rich regions (Behringer and Hall 2016). In *S. cerevisiae*, a comparison between strains carrying the same gene with different GC content revealed that the GC-rich gene had elevated mutation rates resulting from a combination of error-prone DNA polymerase and increased DNA polymerase slippage (Kiktev et al. 2018). Furthermore, regions susceptible to DNA damage via double-strand breaks (DSBs) are GC-rich in prokaryotes (Weissman et al. 2019), potentially revealing an increase in mutational opportunities in genomic regions with high GC content. It is therefore interesting that the eukaryotes with the lowest point mutation rates tend to have AT-rich genomes (Fig. 1): the %GC of *T. thermophila* (22%)*, P. tetraurelia* (28%) and *D. discoideum* (22%) are substantially lower than *C. reinhardtii* (64%), *P. tricornutum* (49%), *D. pulex* (40%), *D. melanogaster* (42%), *S. cerevisiae* (38%), and *C. elegans* (35%), with the exception of *P. falciparum* (20%). While it is possible that eukaryotes with AT-rich genomes have more efficient DNA repair or encounter fewer DSBs, there is not enough data on how GC content relates to large mutations, such as deletions and duplications.

DSBs are generally repaired by one of two pathways: homologous recombination (HR) or non-homologous end joining (NHEJ), which is more error-prone. The DSB repair pathway choice is therefore essential for maintaining genome integrity (Brandsma and Gent 2012), as errors during this repair process can lead to various types of mutations including deletions and duplications (Reams and Roth 2015). Deletions and duplications are widespread mutations that contribute to substantial genomic variation among individuals (Hastings et al. 2009) and are associated with both common and rare diseases (Conrad et al. 2010). Despite their importance and prevalence, gene deletion and duplication rates have been measured using MA experiments in only a few organisms to date. These gene deletion and duplication rates (per gene/generation) are all orders of magnitude greater than point mutation rates (per site/generation): *S. cerevisiae* at 5.5 × 10^–6^ (Lynch et al. 2008), *C. elegans* between 3.4 × 10^–7^ and 1.9 × 10^–6^ (Lipinski et al. 2011; Konrad et al. 2018), *D. melanogaster* at 1.1 × 10^–6^ (Schrider et al. 2013), and *D. pulex* between 3.1 × 10^–6^ and 5.4 × 10^–5^ (Keith et al. 2016; Chain et al. 2019). While organisms with AT-rich genomes have low point mutation rates, it is currently unclear if they also have low gene deletion and duplication rates.

In this study*,* we test whether gene deletion and duplication rates in *Dictyostelium discoideum* are similarly low as is the point mutation rate. *D. discoideum* is a haploid slime mold with an AT-rich genome (78% AT) that is also rich in simple sequence repeats (SSRs), making up over 14% of their 34 Mb genome (Eichinger et al. 2005; Basu et al. 2015; Srivastava et al. 2019). Despite their low point mutation rates, *D. discoideum* has a relatively high indel rate (Kucukyildirim et al. 2020), as does *P. falciparum*, potentially in part because of their AT-rich genome but also their abundance of SSRs, which are susceptible to slippage mutations. *D. discoideum* is resistant to various DNA damaging agents such as UV light, DNA damaging chemicals, ionizing radiation, and gamma rays (Freim and Deering 1970; Deering 1994; Yu et al. 1998; Zhang et al. 2009; Pears et al. 2021), suggesting an enhanced ability to repair DSBs and maintain genome integrity. This organism has a large effective population size and small genome size, which may aid in maintaining relatively high replication fidelity to avoid the accumulation of various types of mutations in their genome (Kucukyildirim et al. 2020).

## Methods

### Copy Number Mutation Identification

The raw sequencing data were accessed from a previously published *D. discoideum* mutation accumulation (MA) experiment (Kucukyildirim et al. 2020). Briefly, two sets of MA lines were cultured in separate labs in Petri dishes, each starting from a single colony and growing continuously in the vegetative single-cell stage. Every two days, a single colony was chosen at random from each MA line to seed a new plate, acting as a single-cell bottleneck. This process was carried out for about 1000 generations in one set, and 2000 generations for the other set. Twenty MA lines from each of the two sets were sequenced, but only 37 reached sequence coverage > 20 × and were kept for analysis (Kucukyildirim et al. 2020). All the raw sequence FASTQ files (Sequence Read Archive Bioproject PRJNA6158150) were trimmed using Trimmomatic with default parameters (Bolger et al. 2014). The tool BWA-MEM (Li 2013) was used for mapping the trimmed reads against the reference genome (NCBI accession number: GCA_000004695.1). The mapped BAM files were used in three copy number variation (CNV) calling programs to identify deletions and duplications that accumulated during the MA experiment: CNVnator (Abyzov et al. 2011), Manta (Chen et al. 2016), and Delly (Rausch et al. 2012). The three CNV calling programs use different signals to identify CNVs, which have shown to provide complementary results (Coutelier et al. 2022). CNVnator calls CNVs using read depth coverage and GC bias correction. Manta uses signals from paired-end sequencing reads by identifying discordant pairs and split reads before performing a local assembly and realignment to call CNVs and breakpoints. Delly calls CNVs using discordantly aligned read pairs and refines the breakends of detected events using split reads, providing breakpoint resolution of CNVs. The program BEDTools v2.30.0 (Quinlan and Hall 2010) was then used to merge CNV information with the *multiIntersect* command to determine the overlapping CNVs among programs and samples, for deletions and duplications separately. Because the detection of CNVs can be improved by combining the calls from multiple programs (Coutelier et al. 2022), all CNVs that passed filtering (see below) were kept for analysis, whether they were called by one, two or three programs.

### Mutation Filtering

CNVs were filtered retaining only mutations occurring in a single MA line that arose during the MA experiment. While this eliminates recurrent deletions and duplications, this approach limits the effects of false positives that result from regions prone to mismapping and uneven coverage. Given *D. discoideum* is haploid, CNVs identified from CNVnator, which bases its CNV calling based on read depth, were only kept if their normalized read depth values corresponded to less than 0.5x (putative deletion) or greater than 1.5x (putative duplication) of the mean read depth. The resulting CNVs from all CNV callers were visually inspected with Integrative Genome Viewer (IGV) (Robinson et al. 2011). All 37 BAM files were indexed and loaded into IGV to show the read sequences and depth of coverage. By comparing across all samples, we were able to ensure that CNV calls were indeed present in a single file through evaluation of the (1) relative depth of coverage, (2) mapping quality, and (3) overall base quality. For the relative depth of coverage, coverage tracks in IGV show how many reads map to different parts of the genome. For CNV calls from CNVnator and Delly, we looked for approximately double the read depth coverage for duplications and zero coverage for deletions. By looking at the flanking regions of the called CNV, we could see whether or not the CNV region was similar throughout the region and flanking region in all other 36 files without the CNV (Suppl. Figure 1). For CNV calls from Manta, we evaluated the number of reads and read pair orientations; for duplications we required at least twice as many reads, and for deletions we required an average of no reads in the CNV, where flanking reads have an insert size spanning the CNV. CNVs meeting these requirements were further filtered by excluding alignments with a mapping quality lower than 20 to prevent false-positive CNVs from poor mapping results. When multiple calling programs called a CNV in the same region, the coordinates from the program that had a better match with the coverage information in IGV were used.

### Overlap of Deletions and Duplications with Genomic Features

To determine genes that were within the putative deletions and duplications, annotated gene coordinates were assessed for overlap with CNVs using BEDTools. A complete gene deletion or duplication was assigned when an entire gene was encompassed in a CNV, while a partial gene CNV was assigned when only a portion of a gene overlapped with a CNV. Only annotated protein-coding genes were kept for calculating mutation rates. The gene deletion and gene duplication mutation rates (μ) were calculated for each MA line using the equation μ = *m*/(*nT*), where m is the number of observed mutations, n is the total number of genes in the genome, and T is the number of generations. Rates were calculated separately for complete gene CNVs and partial gene CNVs. The rate of deletions and duplications per genome per generation were also calculated.

The nucleotide content of CNVs and their breakpoints (50 bp upstream + 50 bp downstream of the estimated breakpoints) was calculated using BEDTools to determine whether GC content differed from the genomic background. The occurrence of simple sequence repeats (SSRs) among CNVs and their breakpoints was also analyzed to test whether CNVs overlapped SSRs more frequently than expected by chance. SSRs were determined using perl scripts (https://cci-git.uncc.edu/wsung/ssrsearch) from a previous analysis of *D. discoideum* SSRs of the same MA samples (Kucukyildirim et al. 2020). All figures and statistical tests were performed in R (R Core Team 2020).

## Results

### Low Rate of Deletion and Duplication in Dictyostelium discoideum

Across all 37 MA lines and an average of ~ 1,500 generations, we identified a total of 18 deletions and 3 duplications (Table 1; Suppl. Table 1). All deletions were complete losses of DNA, and all duplications had more than double the read coverage when compared with other MA lines. Each deletion and duplication was visually verified for read depth and orientation that match CNV expectations in the focal MA line versus all other MA lines (see Methods; Suppl. Figures 1–3**)**. Most MA lines did not have any deletion or duplication (62%; 23 of 37), while 4 MA lines had more than one deletion and/or duplication. The maximum number of CNVs that occurred in a single MA line was four (MA line L54 had four deletions) and one MA line (QS50) had two duplications. The average deletion length was 3,305 bp, and the average duplication length was 4,191 bp. There was no effect of sequencing coverage on the number of deletions and duplications detected among MA lines (Pearson’s *r* = 0.12, *p* = 0.475), and no CNVs were detected in the region on chromosome 2 where duplications are frequently found among strains (Bloomfield et al. 2008). Additionally, the genes found in CNVs (see below) did not have different mean read depth coverage than genes outside of CNVs (*p* = 0.329; mean coverage of 37 × and 36x, respectively).

**Table 1:** Deletions and duplications identified among MA lines by CNV-calling programs

Chromosome	Start	End	Length	Mutation	MA Line	Calling Program
1	1,047,500	1,048,000	500	Deletion	L14	CNVnator
1	4,777,500	4,785,500	8,000	Duplication	QS50	CNVnator
1	4,922,000	4,924,000	2,000	Duplication	QS50	CNVnator
2	693,500	699,500	6,000	Deletion	L56	CNVnator
2	1,555,860	1,556,296	436	Deletion	QS84	Delly
2	4,200,443	4,201,170	727	Deletion	L1	Delly
2	7,157,472	7,159,275	1,803	Deletion	L17	Manta
2	7,186,805	7,193,382	6,577	Deletion	L54	Delly & CNVnator
2	7,258,904	7,259,805	901	Deletion	L54	Delly
3	6,346,000	6,357,000	11,000	Deletion	L25	CNVnator
3	2,931,000	2,935,500	4,500	Deletion	L3	CNVnator
3	1,978,500	1,982,000	3,500	Deletion	L54	CNVnator
4	5,439,500	5,441,500	2,000	Deletion	QS14	CNVnator
5	849,500	853,500	4,000	Deletion	L54	CNVnator
5	3,273,782	3,279,844	6,062	Deletion	QS40	Manta
5	2,608,997	2,610,178	1,181	Deletion	L1	Manta
5	4,803,015	4,805,588	2,573	Duplication	QS40	Manta
5	4,276,322	4,277,619	1,297	Deletion	L52	Manta
5	4,920,500	4,925,000	4,500	Deletion	L53	CNVnator
6	2,886,768	2,887,266	498	Deletion	L1	Delly & Manta
6	3,586,000	3,590,000	4,000	Deletion	QS74	CNVnator

**Fig. 2:**
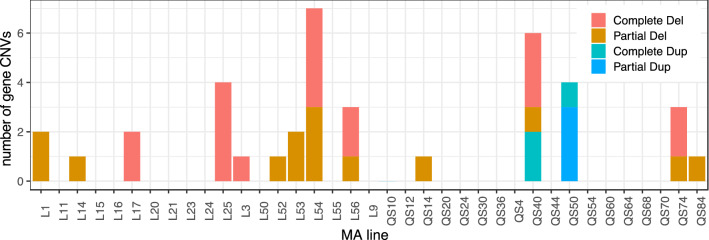
Gene CNV Distribution Across 37 MA Lines. The number of complete and partial gene CNVs per MA line. Gene CNVs are separated by complete gene deletions (red), partial gene deletions (orange), complete gene duplications (blue) and partial gene duplications (aqua)

The genome of *D. discoideum* is gene-dense with about 68% covered by protein-coding genes. All but one of the CNVs overlapped at least one gene (Suppl. Table 2). There was a total of 40 different genes encompassed among the CNVs (including 2 non-coding tRNA genes). Based on random permutations of CNV regions across the genome, the observed number of CNVs overlapping genes (percentile = 0.648) and the number of protein-coding genes in CNVs (percentile = 0.079) were not significantly different than chance alone. Of the 38 protein-coding genes in CNV regions, there were 32 gene deletions (18 complete genes and 14 partial genes), and 6 gene duplications (3 complete genes and 3 partial genes; Fig. 2). The maximum number of protein-coding gene deletions in one MA line was 4 complete gene deletions and 3 partial deletions (L54). There was a maximum of 2 complete gene duplications in MA line QS40 and 3 partial gene duplications in MA line QS50. Altogether, considering protein-coding genes that are either completely or partially overlapping CNVs resulted in an average of 5.11 × 10^–8^ gene deletions and duplications per gene per generation: 3.93 × 10^–8^ gene deletions per gene/generation and 1.18 × 10^–8^ gene duplications per gene/generation (Suppl. Table 1). When considering only complete gene CNVs, the gene deletion and duplication rates were 2.24 × 10^–8^ and 5.91 × 10^–9^ per gene/generation, respectively. The partial gene deletion and duplication rates were similar to complete gene deletion and duplication rates, at 1.69 × 10^–8^ and 5.91 × 10^–9^ per gene/generation, respectively (Suppl. Table 1). Given the low rates recovered compared to other species, we investigated whether these were influenced by conservative CNV filtering (see Methods), in case we filtered out true positives along with our true negatives. We recalculated gene deletion and duplication rates prior to manual filtering, taking all deletions and duplications detected by the three CNV detection tools that were within the same length distribution as our verified deletions and duplications (below 20 kb). This analysis recovered a total of 72 complete gene deletions and 46 complete gene duplications, which resulted in an average of 9.23 × 10^–8^ gene deletions and 5.90 × 10^–8^ gene duplications per gene/generation. We also recalculated gene deletion and duplication rates after excluding reads with low mapping quality (MAPQ < 10) and only retaining genes that had > 20 × average coverage, but this had negligeable effects on our estimates resulting in a slightly higher combined rate of 5.40 × 10^–8^ gene deletions and duplications per gene per generation. We found neither a correlation between the CNV rate in a MA line and its base substitution rate (Pearson’s *r* = − 0.09, *p* = 0.591) nor its indel rate (Pearson’s *r* = 0.06, *p* = 0.715), based on published small-scale mutation rates from the original MA study (Kucukyildirim et al. 2020).

### Copy Number Mutations Preferentially Occur in AT-Rich Regions

**Fig. 3:**
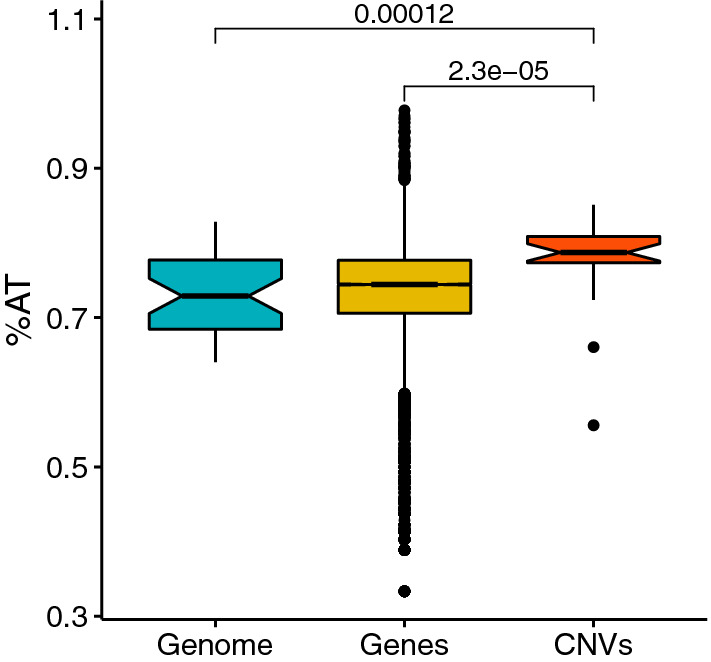
Distribution of %AT across the genome (by chromosome), across genes, and across CNVs. The boxplot notches indicate the median, and Mann–Whitney U-test *p*-values are shown for pairwise tests

As the *D. discoideum* genome is AT-rich, we sought to test whether deletions and duplications occurred in regions with different nucleotide content than the average genomic background. Our expectation was that if CNVs occurred in GC-rich regions, the relatively low GC content in *D. discoideum* could explain the few observed CNVs. In contrast, we found that CNVs were in regions with higher %AT compared to the genomic background (*p* = 1.2e-4) and compared to genes (*p* = 2.3e-5), despite the already AT-rich genome (Fig. 3). This result held if we included 2 kb upstream and downstream of the CNV breakpoints (*p* = 1.3e-4 compared to the genomic background), if we only analyzed 50 bp upstream and downstream of the CNV breakpoints (*p* = 0.006 compared to the genomic background), or if we only included gene CNVs (*p* = 0.016). We also found that CNVs and CNV breakpoints both overlap fewer simple sequence repeats (SSRs) than expected by chance (both *p* < 0.001), in contrast to point mutations that were found to preferentially occur in SSRs (Kucukyildirim et al. 2020).

## Discussion

Mutation accumulation experiments and whole-genome sequencing have advanced our understanding of the rates at which mutations spontaneously arise in eukaryotes. Such studies have helped identify that *D. discoideum* has low point mutation rates: *D. discoideum* has a point mutation rate of around 2.5 × 10^–11^ per site per generation (Saxer et al. 2012; Kucukyildirim et al. 2020), which is considerably lower than rates reported in most other eukaryotes*.* The point mutation rate in *D. discoideum* is even an order of magnitude lower in comparison to organisms with similar effective population sizes like *C. reinhardtii* and *P. tricornutum* (Fig. 1). These two organisms have more than twice the GC content compared to the AT-rich genome of *D. discoideum* (~ 22% GC), raising the possibility that this high AT content contributes to a lower mutation rate. The GC content has been shown to have a notable impact on the probability of mutations (Hodgkinson and Eyre-Walker 2011; Krasovec et al. 2017). In yeast for example, altering the nucleotide sequence but not the amino acid sequence of the *URA3* gene to contain higher GC content led to a sevenfold increase in the rate of mutation and a dependence on the error-prone DNA polymerase (Kiktev et al. 2018). Whether these effects extend to large-scale deletions and duplications remains largely unknown.

In this study, we found low mutation rates of gene deletions and duplications in *D. discoideum*, with a combined estimate of 5.11 × 10^–8^ deletions and duplication per gene per generation. In contrast, other eukaryotes whose rates have been estimated in a similar fashion via mutation accumulation experiments have much higher rates of gene deletion and duplication per gene per generation by orders of magnitude, ranging between 1.3 × 10^–7^ and 7 × 10^–5^ (Lynch et al. 2008; Schrider et al. 2013; Keith et al. 2016; Konrad et al. 2018; Chain et al. 2019). To ensure that the low measured rates were not caused by bioinformatic filtering of true positives, despite our enhanced ability to detect CNVs in haploid cells compared to diploid organisms, we also calculated gene deletion and duplication rates prior to CNV filtering but found similarly low rates that are still an order of magnitude lower than other species. Even when we include partial gene deletions and duplications, the overall gene deletion and gene duplication rates are still lower than other organisms by an order of magnitude. Interestingly, we found that deletions and duplications were underrepresented among SSR regions, which are highly mutable via slippages (Bhargava and Fuentes 2010) and contribute to shaping the mutational profiles of point mutations in *D. discoideum* (Kucukyildirim et al. 2020). Despite their unusually SSR-rich genome (Srivastava et al. 2019), *D. discoideum* also has a very low microsatellite mutation rate, perhaps due to the evolution of efficient repair mechanisms (McConnell et al. 2007).

The overall low rate of mutations in *D. discoideum* is consistent with observations that this organism is highly resistant to DNA damage (Pears et al. 2021). *D. discoideum* is known to withstand various DNA damaging agents, including ionizing radiation, which induce DNA strand breaks (Freim and Deering 1970; Zhang et al. 2009). It has been proposed that this high genome stability stems from effective DNA repair mechanisms, which might be vital in *D. discoideum* (Deering 1994). Spontaneous mutations can occur via error-prone DNA polymerases (Makarova and Burgers 2015), so the pathway used to repair DNA breaks can influence the probability of mutation and overall genome stability (Hsu et al. 2011). In *D. discoideum,* the knock down of NHEJ proteins has little to no impact during growth in tolerance to agents that induce DSBs (Hudson et al. 2005). This means that the less error-prone HR repair pathway is likely used to repair DNA damage and thus can moderate genome stability, possibly explaining the low deletion and duplication rates.

It is possible that the *D. discoideum* genome avoids the accumulation of large deletion and duplication mutations in addition to point mutations and microsatellite repeats thanks to its reported strong DNA repair mechanisms and replication fidelity. But it might also have fewer opportunities for spontaneous mutations due to its low GC content that plausibly confers lower vulnerability to DNA damage and recombination. High genomic GC content has been associated with an increase in DSBs (Weissman et al. 2019) and some types of recombination like break-induced replication, which is a unique type of homologous recombination that repairs single-ended DSBs and subsequently can lead to higher levels of mutation (Malkova and Haber 2012; Kiktev et al. 2018). In *S. cerevisiae*, GC-rich regions of the genome are correlated with higher rates of meiotic recombination, which makes them more susceptible to mutation as a consequence of DNA polymerase slippage (Makarova and Burgers 2015; Kiktev et al. 2018). In *D. discoideum*, we found that gene deletions and duplications were AT-rich even compared to the AT-rich genome background, in contrast to expectations that GC content is associated with more mutations or DNA breaks. Unless *D. discoideum* has greater efficiency in repairing the DNA breaks in GC-rich regions, we did not detect a direct relationship between the AT-rich genome of *Dictyostelium* and its low rates of deletions and duplications.

## Conclusion

The genetic mechanisms underlying CNVs can differ amongst organisms for various reasons. DNA damage can be repaired through various response pathways where one might be more error-prone to another, like non-homologous end joining compared to homologous recombination. Having a genomic content that increases the vulnerability to DNA damage or relies on error-prone pathways can influence mutation rates. In the case of GC-rich microbial eukaryotes, it is likely that they are more liable to mutation and recombination (Vinogradov and Anatskaya 2017), and therefore possibly account for the higher mutation rates experimentally observed. The lower mutation rates in *D. discoideum*, including of gene deletions and duplications as we report here, can potentially result from a combination of lower DNA damage and higher efficacy of DNA repair machinery (Sniegowski et al. 2000; Baer et al. 2007). It would be interesting to further delve into an organism with a similar effective population size but high GC content, like *P. tricornutum* or *C. reinhardtii* to establish whether these factors influence the organism’s ability to maintain genome stability and low mutation rates.

## Supplementary Information

Below is the link to the electronic supplementary material.

Example view in IGV of a (A) deletion and (B) duplication called by CNVnator, with the CNV region highlighted in the red box. Supplementary file1 (PDF 1907 KB)

Example view in IGV of a deletion called by Delly, with the CNV region highlighted in the red box. Supplementary file2 (PDF 2594 KB)

Example view in IGV of a (A) deletion and (B) duplication called by Manta, with the CNV region highlighted in the red box. Supplementary file3 (PDF 1247 KB)

Supplementary file4 (XLSX 23 KB)
