# Supplementary material for: Very Low Rates of Spontaneous Gene Deletions and Gene Duplications in Dictyostelium discoideum
Source: J Mol Evol. 2022 Dec 9;91(1):24–32. doi: 10.1007/s00239-022-10081-1 (PMC9849192; doi:10.1007/s00239-022-10081-1)

A) Deletion called by CNVnator
Chromosome 2:693,500-699,500 in MA line L56 (SRR11509897)

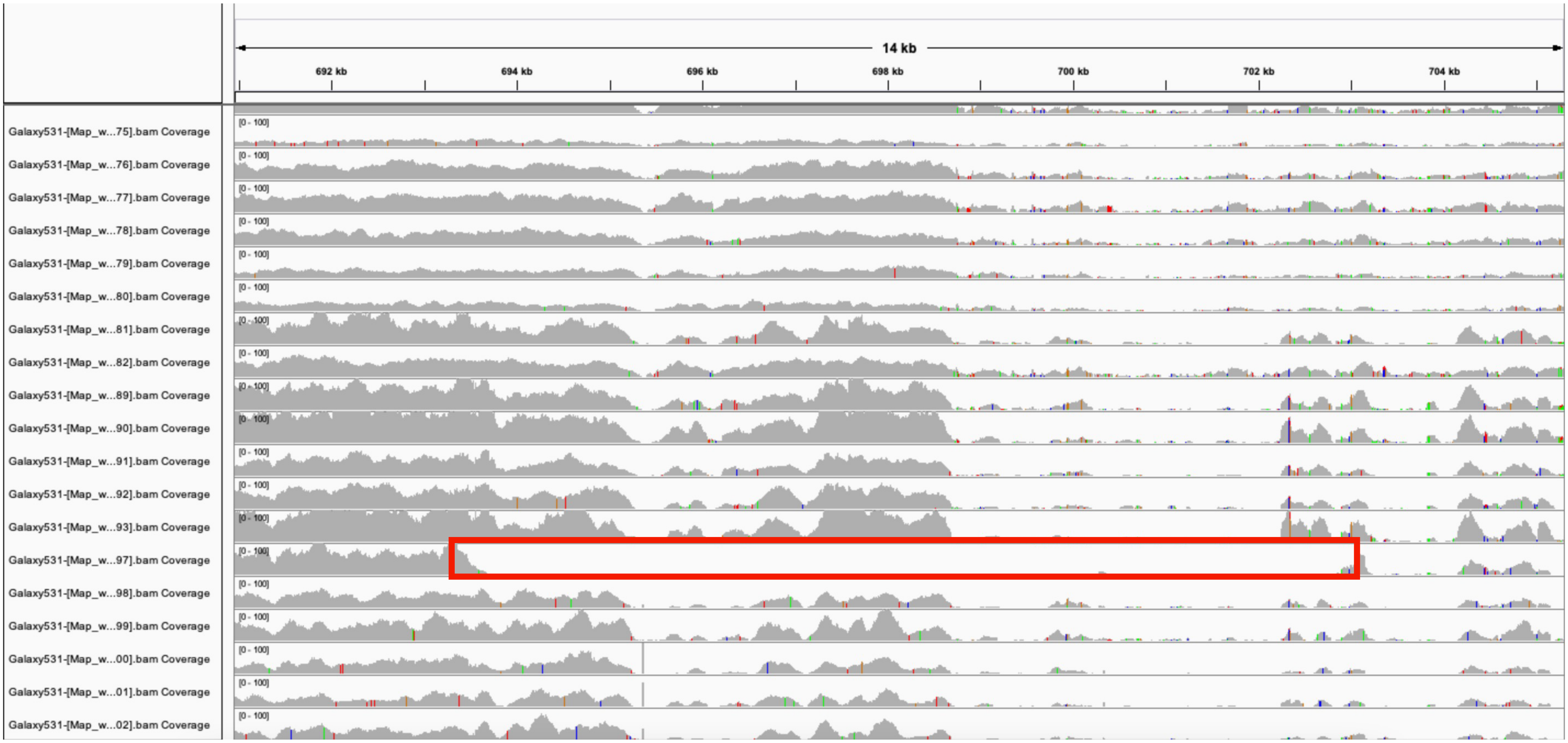

B) Duplication called by CNVnator
Chromosome 1:4,777,500-4,785,500 MA Line QS50 (SRR11433368)

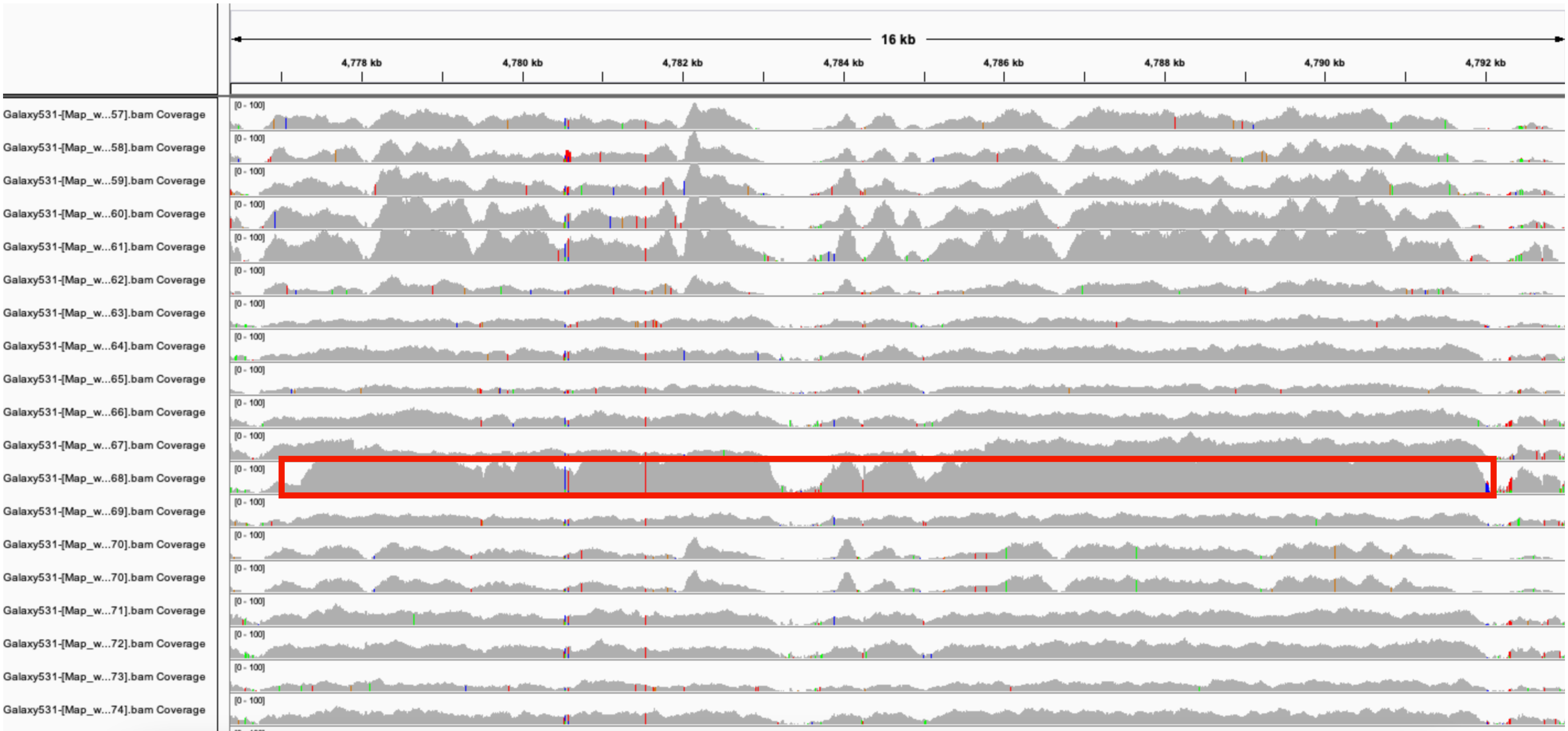

Supplement: Supplementary file 1 — Example view in IGV of a (A) deletion and (B) duplication called by CNVnator, with the CNV region highlighted in the red box. Supplementary file1 (PDF 1907 KB) [file 239_2022_10081_MOESM1_ESM.pdf]
