# Supplementary figures and images for: Very Low Rates of Spontaneous Gene Deletions and Gene Duplications in Dictyostelium discoideum
Source: J Mol Evol. 2022 Dec 9;91(1):24–32. doi: 10.1007/s00239-022-10081-1 (PMC9849192; doi:10.1007/s00239-022-10081-1)

# Deletion called by Delly

## Chromosome 2:7,186,805-7,193,382 in MA line L54 (SRR11509899)

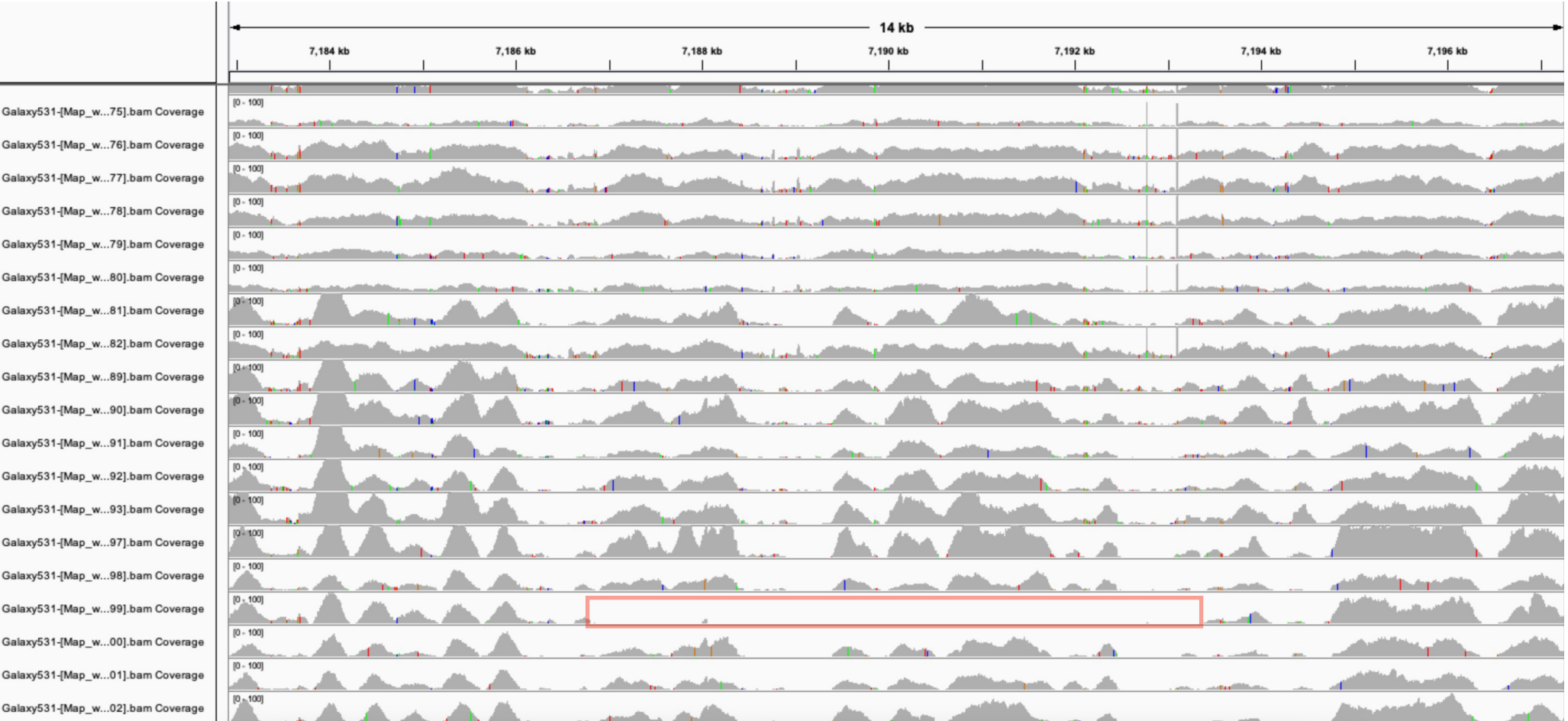

Supplement: Supplementary file 2 — Example view in IGV of a deletion called by Delly, with the CNV region highlighted in the red box. Supplementary file2 (PDF 2594 KB) [file 239_2022_10081_MOESM2_ESM.pdf]
