# Supplementary material for: Very Low Rates of Spontaneous Gene Deletions and Gene Duplications in Dictyostelium discoideum
Source: J Mol Evol. 2022 Dec 9;91(1):24–32. doi: 10.1007/s00239-022-10081-1 (PMC9849192; doi:10.1007/s00239-022-10081-1)

A) Deletion called by Manta
Chromosome 2:7,157,472-7,159,275 MA Line L17 (SRR11433359)

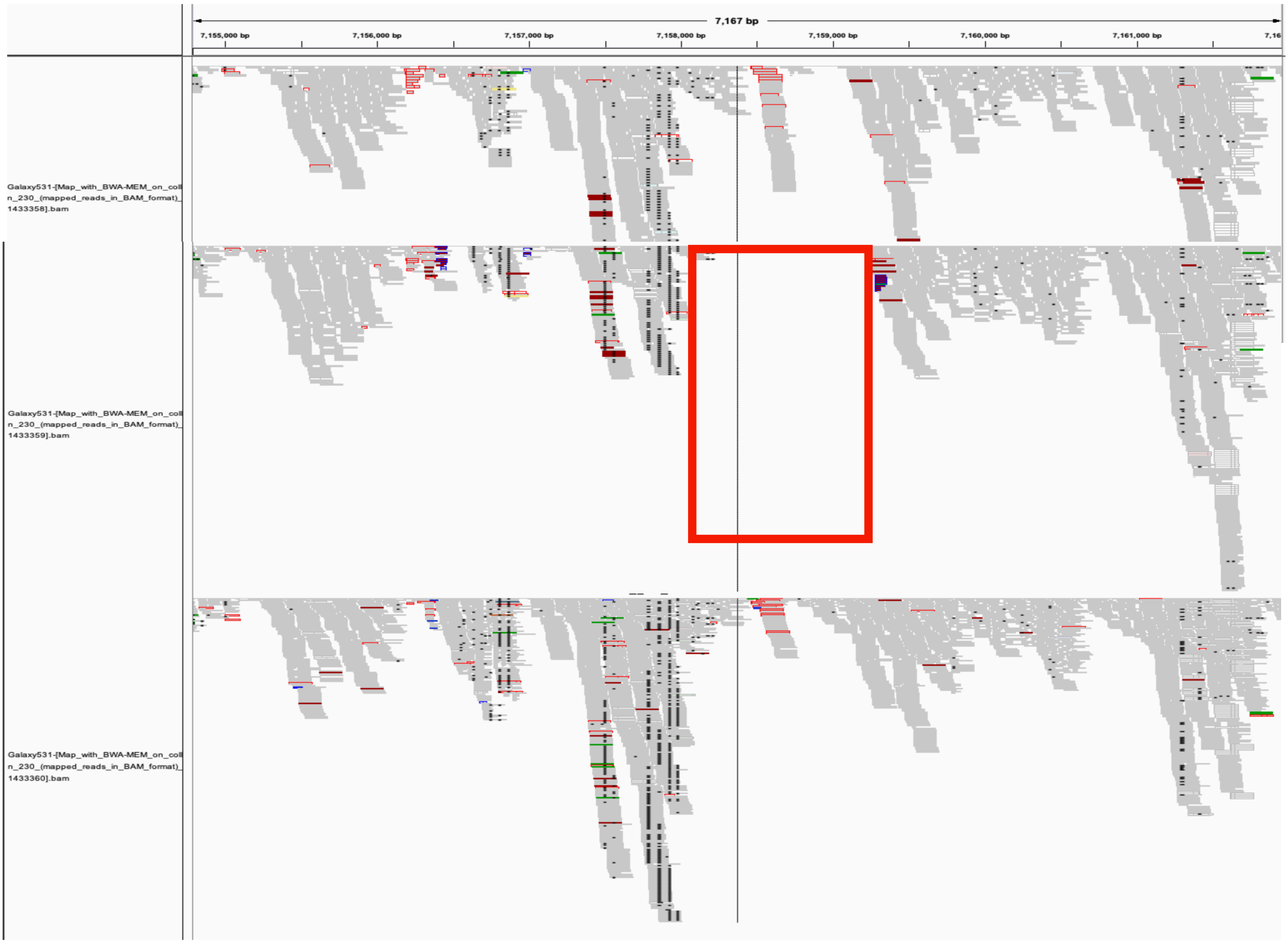

B) Duplication called by Manta
Chromosome 5:4,803,015-4,805,588 MA Line QS40 (SRR11433365)

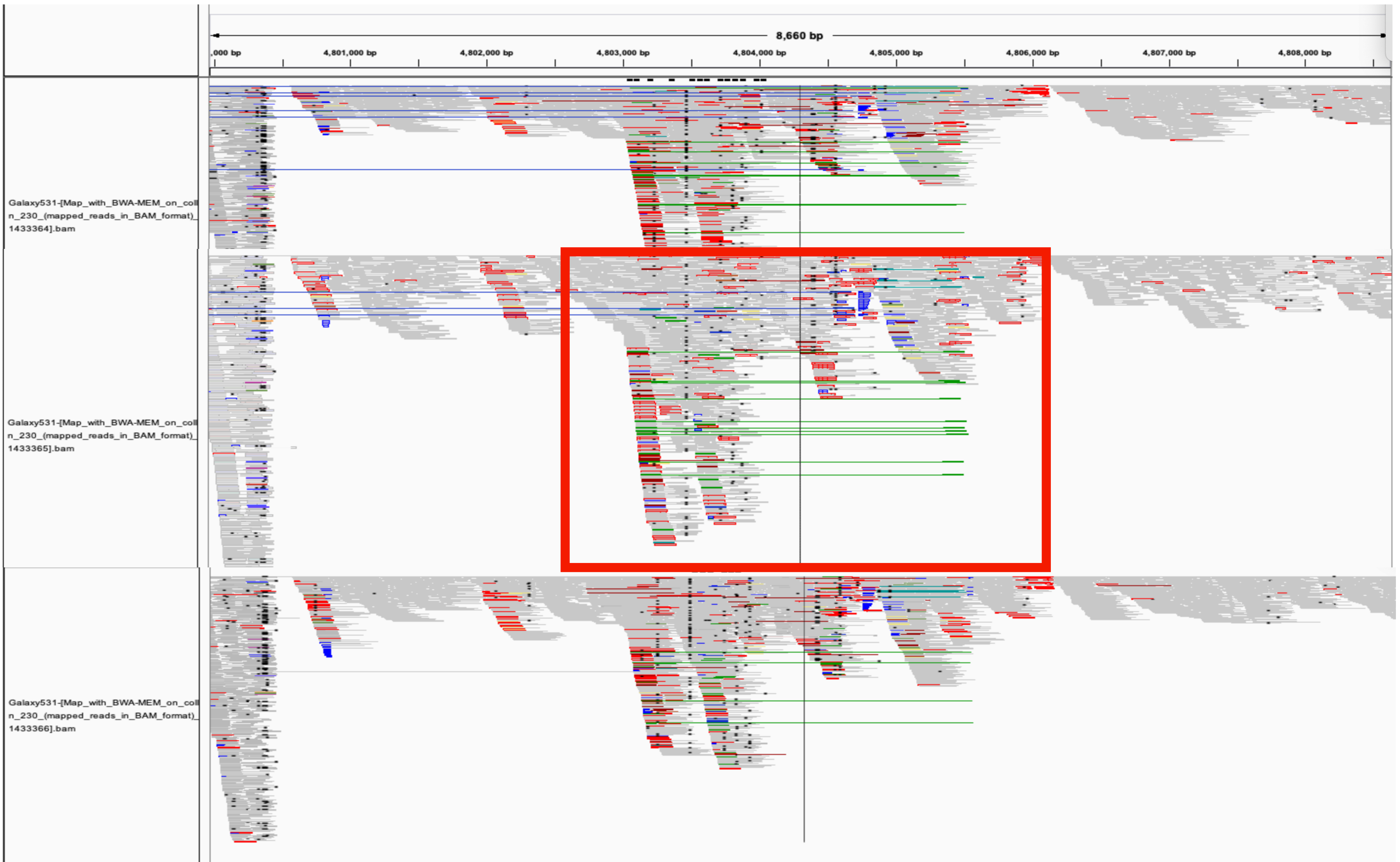

Supplement: Supplementary file 3 — Example view in IGV of a (A) deletion and (B) duplication called by Manta, with the CNV region highlighted in the red box. Supplementary file3 (PDF 1247 KB) [file 239_2022_10081_MOESM3_ESM.pdf]
